# A novel variant of *TFAP2A* in a familial case of branchio-oculo-facial syndrome: Insights from structural bioinformatics and molecular dynamics simulation

**DOI:** 10.1016/j.gendis.2025.101641

**Published:** 2025-04-15

**Authors:** Jingjing Zhao, Nuo Si, Ningbei Yin, Tao Song

**Affiliations:** a The Cleft and Palate Department of Plastic Surgery, Plastic Surgery Hospital, Chinese Academy of Medical Sciences, Peking Union Medical College, 33 Badachu Road, Shijingshan District, Beijing, 100144, China; b Research Center of Plastic Surgery Hospital, Chinese Academy of Medical Sciences & Peking Union Medical College, Beijing 100144, China

Branchio-oculo-facial syndrome (BOFS; OMIM 113620) is an autosomal dominant condition characterized by three main features, respectively: branchial defects, ocular anomalies, and craniofacial defects. BOFS is a distinctive multiple congenital anomaly syndrome with variable severity.^1^

The *TFAP2A* gene, located on chromosome 6p24 and consists of seven exons encoded transcription factor AP2A with 437 amino acids (NM_001042425). *TFAP2A* is currently the only known causative gene. AP-2A contains three conserved domains: a proline and glutamine rich (PG) domain in the N-terminal region, which is responsible for transcriptional activation; a central basic DNA binding (B) domain; and a highly conserved helix-span-helix (HSH) domain in the C-terminal region, which interferes with dimerization and site-specific DNA binding.^1^, ^2^, ^3^, ^4^ AP-2A binds the consensus sequence 5′-GCCNNNGGC-3'. AP-2A functions as either a homodimer or as a heterodimer with similar family members. AP-2A activates the transcription of some genes while inhibiting the transcription of others.

In this study, 5 members of a Chinese family were analyzed. Two affected individuals in two generations that includes a male aged 10 (III-1) and a female aged 34(II-2). (II-2) and (III-1) were diagnosed with BOFS. The pedigree of the family (Fig. 1A). (II-2) hair started to turn gray at the age of 30 years. The first child was miscarried at the age of 7 months, intrauterine distress, axillary and vulvar hair was absent, hair volume did not show significant thinning, history of thick lip thinning surgery. Bilateral auricular deformities, history of purulent discharge from the right preauricular fistula, which was treated surgically, right second-gill slit fistula, pseudo-labial cleft, complained of convex mouth and short midriff in her youth, that appearance improved with age (Fig. 1B).

**Figure 1:**
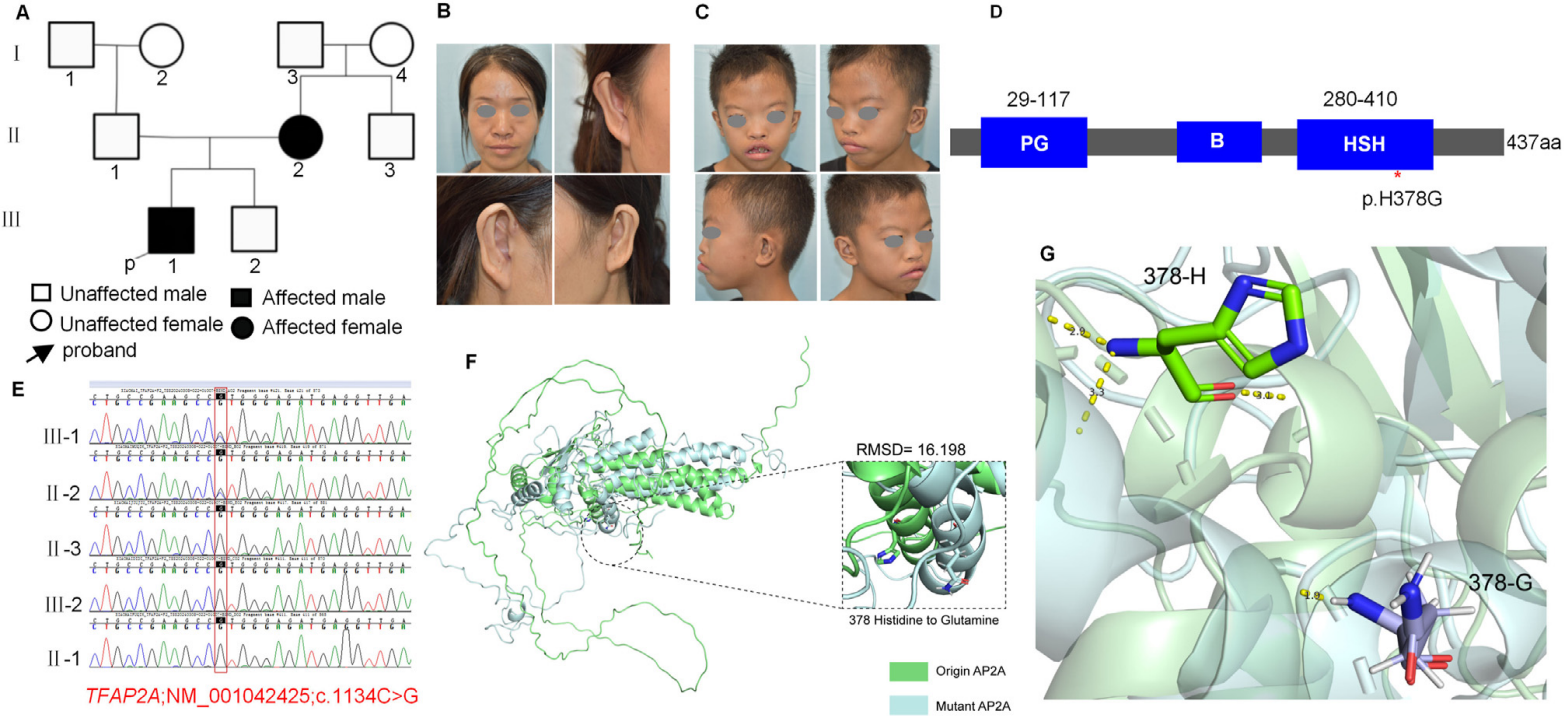
Overview of the Work Scheme Conducted in This Study. (A) The pedigree of the Chinese family with BOFS shows an autosomal dominant pattern of inheritance. Whole genome sequencing of 5 members was performed and the proband was indicated by an arrow (B) II-2 demonstrated typical pseudocleft and bilateral auricular deformity. (C) Demonstrated typical pseudocleft, more hair on the forehead, thick and connected eyebrows, wide eye spacing, upward slanting eye slits, horizontal strabismus, collapsed nose, short philtrum, buck teeth, convex mouth deformity, bowed upper lip, low-set ears with excessive postauricular rotation(D) A novel mutation in *TFAP2A*. Results of genetic Sanger validation in the neonatal BOFS case. The variant c.1134 C > G; (p.His378Gln) was found in the TFAP2A gene on chromosome in patients (III-1,II-2). Fig. 1D is not drawn to scale. (E) The protein structure of the TFAP2A, PG proline, and glutamine-rich domain; B, basic DNA binding domain; HSH, helis span helix domain. The mutation in this study is indicated by a red asterisk(F) The 3D structure of human origin AP2A and mutant H378G. RMSD of origin AP2A and H378G.(G) The hydrogen bonds are shown in yellow dotted lines and the numbers on the hydrogen bonds represent the length in units of Ångström (Å), where 1 Å equals 0.1 nm (nm). It can be observed that both the number and the length of the hydrogen bonds are altered by the mutation.

(III-1) was the product of a non-consanguineously married couple. A young male infant was born at 32 weeks of gestation and subsequently admitted to the neonatal intensive care unit due to respiratory distress syndrome and low birth weight (2200 g). Detailed clinical examination revealed left overfolded ear and Bilateral nasolacrimal duct stenosis was surgically treated at the age of 5. Currently, the right eyelid closure is incomplete with tearing. (III-1) was the second child of non-consanguineous Asian parents. On examination, the boy had unilateral auricular deformity (low-set ears); Strabismus; tooth abnormalities, Short philtrum (protruding teeth and lips); lip (prominent philtral pillars); broad nasal bridge; thick upper lip with prominent vertical ridges of the philtrum, malformed nose with broad bridge and flattened tip. His height was 135 cm and his weight 28 Kg. The boy did not have hemangioma. No postauricular skin scarring lesions, second-gill slit fistula, mucous secretion on extrusion, no scalp cysts less than 2 mm in diameter, no abnormalities on cardiopulmonary auscultation, abdominal tenderness, and no obvious enlargement of the liver or spleen. There were no syndactyly or polydactyly, the muscle tone of the limbs was grade 5, and the neurological examination showed no abnormality. There was no communication disorder, no significant hearing impairment, and no abnormality on fundus examination (Fig. 1C). The maternal and paternal grandparents of the proband (I1–I4)do not exhibit any malformations of the ears or lips, nor have any related clinical manifestations been observed.

The causative gene in the family was investigated through whole genome sequencing of the 5 individuals (II-1,II-2,II-3, III-1 and III-2). The proband was diagnosed with BOFS with a novel heterozygous likely pathogenic variant (c.1134 C > G; p. His378 Gln) in exon 7 of the *TFAP2A* (Fig. 1E). This gene was sequenced from the family 5 members by Sanger sequencing (Fig. 1D), which showed that the proband and his mother (III-1 and II-2) are heterozygous carriers of the *TFAP2A* variant and other unaffected members (II-1,II-3, III-2) are wild type (WT). This TFAP2A variant (c.1134 C > G; p. His378 Gln) was not found in public databases (gnomAD, 1000 Genomes Browser, and HGMD); thus, it is a novel mutation and is likely to be pathogenic. The primer sequences for *TFAP2A*-F2 (TCACGGCCTGTTCTGTTCTC) and *TFAP2A*-R2 (TCTCTGCTCCACTTGTGCTG, 598bp).

Our reported family showed no significant clinical presentation similarity from another with an H384Y exon 7 mutation.^1^^,^^5^ with a missense H384Y mutation in exon 7. *TFAP2A* genotyping in BOFS patients doesn't predict a specific phenotype, and no mutation-specific genotype–phenotype correlation is evident yet; more studies are needed.^1^

The structures of WT and mutated (H378G) human AP2A were predicted in Gromacs. The AP2A protein AlphaFold structure was obtained from the Uniprot database. Utilizing the powerful capabilities of PyMOL, I mutated the histidine residue at position 378 of the AP2A protein to glutamine. Subsequently, the Gromacs software (2022.5 released) was used to perform molecular dynamics simulations on the mutated protein structure, encompassing the processes of energy minimization, NVT canonical ensemble, and NPT isothermal-isobaric ensemble. Root Mean Square Deviation (RMSD), a widely used quantitative parameter for comparing differences between protein structures. An RMSD value below 2 Å typically signifies a minor deviation, reflecting negligible structural alterations in proteins. Conversely, a substantial rise in RMSD, reaching 4–5 Å or higher, may imply significant conformational shifts within the molecule, encompassing changes in protein secondary structure, ligand displacement, or active site rearrangement. Notably, the RMSD value obtained from comparing the protein structure before and after the mutation was 16.198 Å, indicating a significant impact of the modification on the protein's structure. The illustrations of the protein structures before and after mutation, as shown in (Fig. 1F and G), provide a visual representation of these structural alteration. The hydrogen bonds are shown in yellow dotted lines and the numbers on the hydrogen bonds represent the length in units of Ångström (Å), where 1 Å equals 0.1 nm (nm). It can be observed that both the number and the length of the hydrogen bonds are altered by the mutation. The Materials and methods are detailed in the Supplementary Data.

By combining the clinical characteristics, genetic mode, and gromacs analysis results, we propose that variant (c.1134 C > G; p. His378 Gln) in exon 7 of the *TFAP2A* gene was likely causative variant for the features of the Chinese family.

## CRediT authorship contribution statement

**Jingjing Zhao:** Data curation, Formal analysis, Methodology, Writing – original draft, Writing – review & editing. **Nuo Si:** Methodology, Supervision. **Ningbei Yin:** Conceptualization, Investigation, Supervision, Writing – review & editing. **Tao Song:** Conceptualization, Supervision, Writing – review & editing.

## Data availability

All data supporting the findings of this study are available within the article and its Supplementary Materials.

## Conflict of interests

The authors have no competing interests to declare.
