# Supplementary material for: A novel variant of TFAP2A in a familial case of branchio-oculo-facial syndrome: Insights from structural bioinformatics and molecular dynamics simulation
Source: Genes Dis. 2025 Apr 15;12(6):101641. doi: 10.1016/j.gendis.2025.101641 (PMC12281163; doi:10.1016/j.gendis.2025.101641)
Supplement: Multimedia component 1 [file mmc1.docx]

Supplementary Data for " A novel variant of TFAP2A in a familial case of Branchio-oculo-facial syndrome:Insights from structural bioinformatics" （GENDIS 101641）

Materials and methods

Subjects

Two patients from one family diagnosed with BOFS by genetic testing in our hospital were selected as the research subjects. After the informed consent of the patients and their families, the families were investigated. Peripheral blood samples (2 mL each) were collected from family members into EDTA-containing tubes for anticoagulation.The study was approved by the ethical committee of Plastic Surgery Hospital (Beijing, China).

Whole genome sequencing (WGS)

Genomic DNA was extracted from the peripheral blood using a magnetic blood genomic DNA kit (Tiangen, China) following the manufacturer’s instructions. DNA was fragmented to an average size of 350 bp and used to create a DNA library following established Illumina paired-end protocols. The Illumina Novaseq 6000 platform (Illumina, Inc, San Diego, CA) was used for enomic DNA sequencing following standard protocols by Novogene Bioinformatics Technology Co. Ltd (Beijing, China).

Sanger Sequencing

Sanger sequencing was conducted for the c.1134C>G mutant and the wild-type (WT) *TFAP2A* gene. The primer sequences used for *TFAP2A*-F2 were TCACGGCCTGTTCTGTTCTC, and for *TFAP2A*-R2, they were TCTCTGCTCCACTTGTGCTG, resulting in an amplicon size of 598 bp.

Molecular Dynamics Simulation

The structures of the wild-type (WT) and mutated (H378G) human AP2A were predicted using Gromacs. The AP2A protein AlphaFold structure was acquired from the Uniprot database. PyMOL was utilized to mutate the histidine at position 378 of the AP2A protein to glutamine. Molecular dynamics simulation of the mutated protein structure was conducted using Gromacs software (version 2022.5)^6^, following these stages: energy minimization, NVT canonical ensemble, and NPT isothermal-isobaric ensemble. During energy minimization, atomic positions were adjusted to minimize the potential energy of the system, ensuring a stable starting point for the simulation. In the NVT canonical ensemble stage, the system was simulated under constant volume and temperature conditions, allowing for the exploration of accessible microstates at a fixed temperature and volume. The final stage involved simulating the system under constant temperature and pressure conditions (NPT ensemble), which is crucial for understanding the protein's behavior in a physiologically relevant environment. By following these steps, insights into the structural and dynamical properties of the mutated AP2A were gained, which could inform our understanding of its function and potential interactions with other biomolecules.
